# A Full Polymer Piezoelectric Flextensional Energy Harvester

**DOI:** 10.3390/mi17080955

**Published:** 2026-08-12

**Authors:** Nadia Ahbab, Sidra Naz, Bingqi Zhao, Tian-Bing Xu

**Affiliations:** Department of Mechanical and Aerospace Engineering, Old Dominion University, Norfolk, VA 23529, USA; nahba001@odu.edu (N.A.); snaz@odu.edu (S.N.); bzhao004@odu.edu (B.Z.)

**Keywords:** piezoelectric PVDF polymer, force amplification, PLA, additive manufacturing, energy harvesting, flextensional frame, low-frequency vibration, energy storage

## Abstract

This study presents a full polymer piezoelectric flextensional energy harvester (FPPFEH) comprising a single-layer poly(vinylidene fluoride) (PVDF) film bonded to a 3D-printed polylactic acid (PLA) flextensional frame. For an arm inclination angle of θ=10°, the free-body model gives a theoretical geometric force-amplification factor of $M_{F}=\cot\theta \approx 5.67$; this value represents an ideal upper bound and was not independently validated by local force or strain measurements. During assembly, the film was tensioned only to remove visible slack and maintain a flat configuration. No intentional pretension was applied, and any residual tension was not measured. Off-resonance force-controlled tests showed that the generated voltage was approximately proportional to the dynamic input force and nearly independent of frequency after accounting for attenuation caused by the finite measurement-input impedance. The ideal quasi-static model overpredicted the absolute voltage by a nearly constant factor across the tested force range. This offset is consistent with a lumped reduction associated with frame compliance and the in-plane anisotropy of the PVDF film, neither of which was independently measured. At 30Hz and $12.32\;N_{\mathrm{rms}}$, the rectified output charged a 6600μF supercapacitor to 2.10V in 14min, corresponding to 14.55mJ of stored energy. Under base-acceleration excitation from 0.05 g to 1 g, the voltage peak occurred between 112.88 and 116.49Hz, close to the electrical anti-resonance near 114Hz, and reached $12.11\;V_{\mathrm{peak}}$ at 1 g. Near resonance, the highest measured power among the tested resistive loads occurred between 150 and 200kΩ; however, the exact optimal resistance could not be resolved from the four tested loads. These results demonstrate off-resonance force-driven energy storage and resonance-mode vibration energy harvesting within the tested conditions.

## 1. Introduction

Self-powered electronic systems increasingly rely on energy harvesting to convert ambient mechanical inputs into electrical power for sensing, monitoring, and low-power electronics [1,2,3,4,5,6]. Many target environments including wearable devices, wireless sensor networks, and distributed structural monitoring systems provide excitation that is low-frequency, broadband, and with irregular characteristics. In these conditions, conventional batteries impose maintenance burdens and lifetime limitations that constrain long-term deployment scalability [7,8,9,10,11].

Piezoelectric energy harvesting is particularly attractive for such applications due to its direct electromechanical conversion, compact form factor, and solid-state reliability [1,2,3,4]. Among available piezoelectric materials, poly(vinylidene fluoride) (PVDF) and its copolymers offer a compelling combination of low density, mechanical flexibility, and fatigue resistance under cyclic deformation, enabling conformal and large-strain implementations that are difficult or impossible to achieve with brittle piezoelectric ceramics [7,8,9,12,13,14].

A persistent limitation of PVDF-based harvesters, however, is their relatively low electromechanical coupling coefficient and charge density compared with piezoceramics, which reduces electrical output under modest excitation levels [2,9,15,16,17]. Numerous mechanical strategies have been introduced to overcome this limitation by increasing the effective strain transferred into the active film layer, including cantilever beam architectures, nonlinear and bistable mechanisms, frequency up-conversion schemes, and geometric force- or displacement-amplification structures [18,19,20,21,22,23,24,25].

Flextensional-based force-amplification frames are particularly well suited to PVDF applications because they enhance axial strain through geometric leverage rather than relying exclusively on resonance tuning [17,26,27,28]. By redirecting an applied transverse compressive load into amplified axial tension at the film endpoints, these frames increase the effective stress in the piezoelectric element while preserving the ability to operate in off-resonant, quasi-static conditions, an important practical advantage in environments where the excitation frequency is variable or unpredictable [29,30,31,32,33]. However, most reported force-amplification designs employ metallic frames or simultaneously optimize multiple geometric parameters, which increases fabrication complexity and system mass and makes it difficult to isolate the contribution of individual design variables to overall harvester performance [34,35,36,37,38,39].

Additive manufacturing offers a practical route to lightweight compliant amplification structures with rapid design iteration and high geometric freedom. Polylactic acid (PLA) is widely used in fused-deposition modeling (FDM) due to its manufacturing accessibility and dimensional stability [12,40]. Beyond these practical advantages, the choice of PLA as the frame material carries specific scientific motivation. FDM-printed PLA exhibits a Young’s modulus of approximately 2.5–3.5 GPa along the print direction [41,42], compared to 70–200 GPa for the aluminum or steel frames employed in prior metallic flextensional harvesters [34,35,36,37,38,39]. This substantially lower modulus enables larger elastic arm deflection per unit input force, which is beneficial when the transduction element is a thin, flexible PVDF film sensitive to stress concentrations at bonded interfaces. Furthermore, the reduced frame stiffness relative to metallic alternatives allows a greater proportion of the applied input displacement to manifest as axial strain in the PVDF active region, rather than being absorbed within the frame structure itself. Although polymer frames have been combined with piezoelectric materials for customizable harvesting architectures [27,43,44,45,46], systematic experimental studies that quantify force-amplification behavior specifically in 3D-printed polymer frames integrated with PVDF films remain limited.

In this paper, a lightweight full polymer piezoelectric flextensional energy harvester (FPPFEH) that bonds a single-layer piezoelectric PVDF film to a 3D-printed PLA flextensional frame is systematically investigated. The FPPFEH device design and theoretical descriptions are illustrated in Section 2. Section 3 provides the details of the FPPFEH prototype fabrication and comprehensive characterization methods. The results and discussions of the FPPFEH are presented in Section 4. Finally, the work is summarized in Section 5.

## 2. FPPFEH Design and Theoretical Description

### 2.1. Design and Working Principle

**Figure 1:**
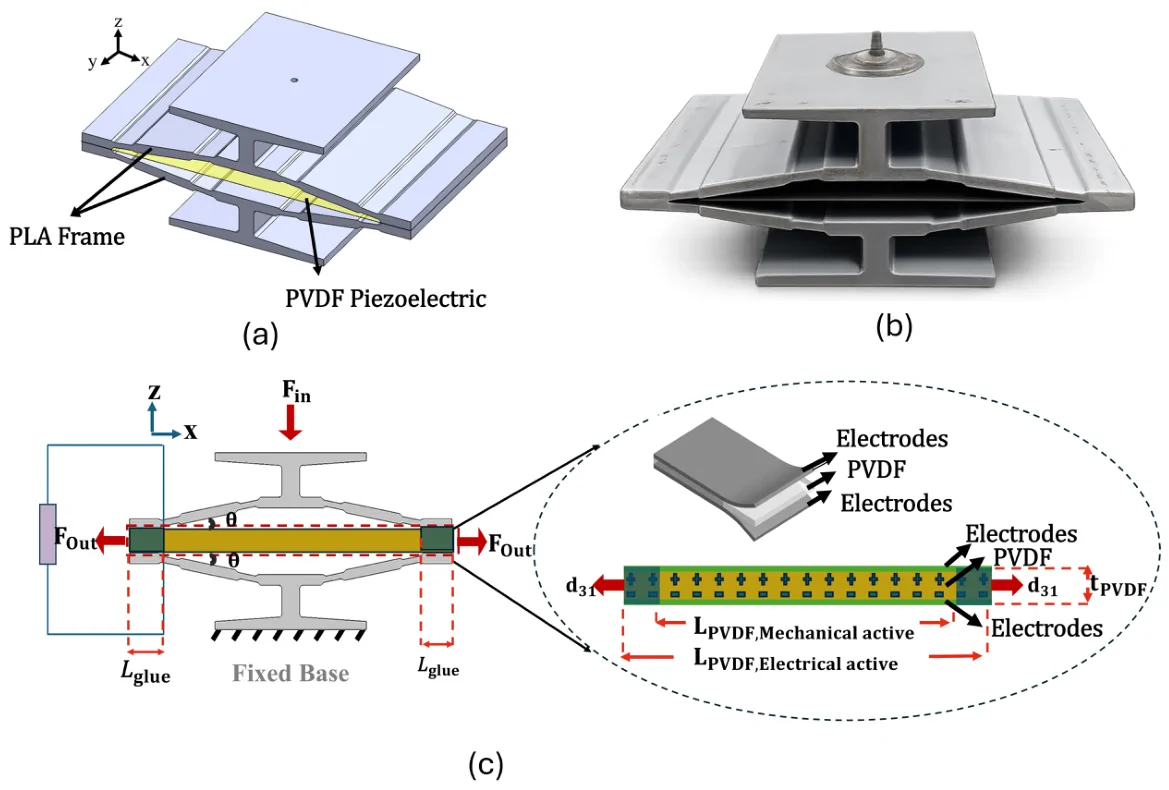
Full polymer piezoelectric flextensional energy harvester (FPPFEH). (**a**) PLA frame and bonded PVDF film. (**b**) Fabricated prototype. (**c**) Working principle and active PVDF regions.

The FPPFEH comprises a single-layer poly(vinylidene fluoride) (PVDF) film bonded to a 3D-printed polylactic acid (PLA) flextensional frame. As shown in Figure 1, the frame consists of two mirrored halves. In the upper half, a central loading platform is stiffened by an I-beam, which provides a measure of flexure, and a web transmits the load to a pair of inclined arms that extend outward and downward toward the two end caps. The lower half mirrors this arrangement, with arms running from the same end caps down to the fixed base. The PVDF film spans the two end caps, so that each cap is loaded simultaneously by one upper and one lower arm, while the PVDF film spans the two end caps. The arm inclination angle θ, measured from the horizontal *x*-axis, was set to 10° for the prototype examined here.

The PVDF film is bonded to the frame only at its two end regions, leaving the central span free to deform. Two active regions are therefore distinguished. The mechanically active region is the unbonded central span that undergoes most of the axial deformation and generates most of the piezoelectric charge. The electrically active region extends over the full electrode-covered area and determines the device capacitance. The dimensions of these regions are reported in Section 3.1.

**Figure 2:**
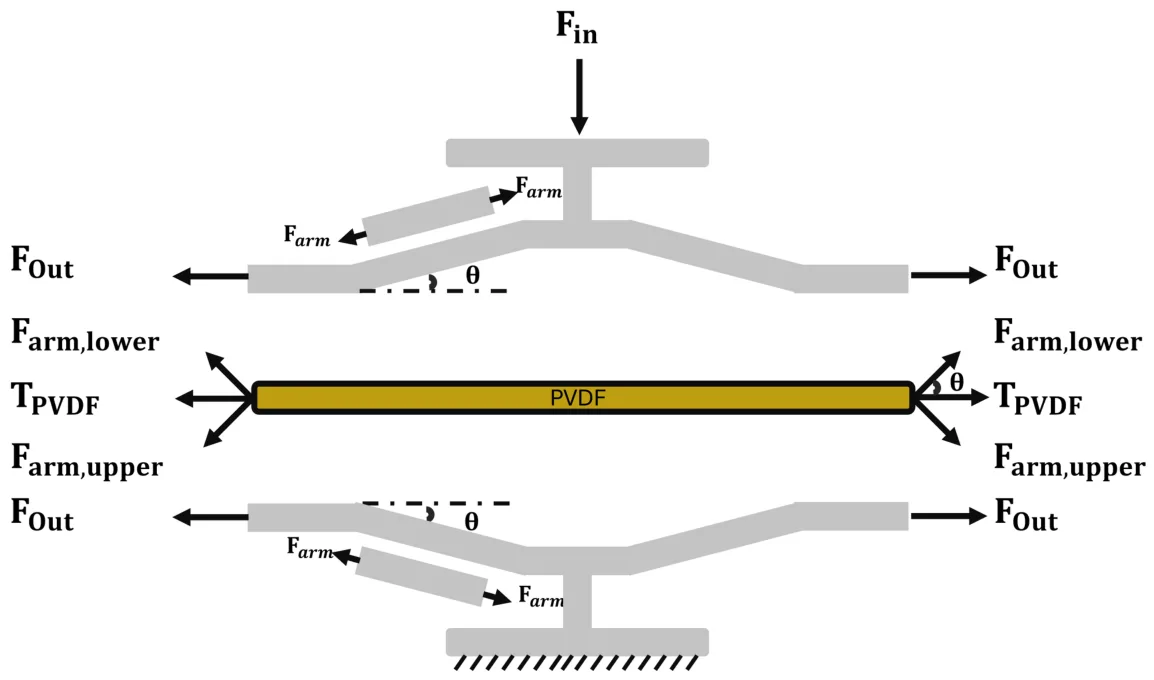
Free-body diagram of the FPPFEH showing the input force, arm forces, output forces, and PVDF tension.

A vertical compressive force $F_{\mathrm{in}}$ applied to the central loading platform is redirected into axial forces along the inclined arms, as illustrated in Figure 2. Under symmetric quasi-static loading, each upper arm carries an axial force of magnitude $F_{\mathrm{arm}}$. Because the loading platform is connected to the two upper arms, vertical equilibrium of the upper platform gives

(1) $$F_{\mathrm{in}}=2F_{\mathrm{arm}}\sin\theta .$$

At each end cap, an upper and a lower arm act together. Their vertical components oppose and cancel one another, whereas their horizontal (x-direction) components share the same direction and combined. The outward force on each bonded film end, which equals the tension carried by the film, is therefore

(2) $$F_{\mathrm{PVDF}}=2F_{\mathrm{arm}}\cos\theta .$$

Here, $F_{\mathrm{PVDF}}$ denotes the magnitude of the internal tensile force carried by the PVDF film.

Combining Equations (1) and (2) gives the theoretical geometric force-amplification factor [28,43]

(3) $$M_{F}=\frac{F_{\mathrm{PVDF}}}{F_{\mathrm{in}}}=\cot\theta .$$

At θ=10°, the ideal geometric amplification factor is

(4) $$M_{F}=\cot\left(10{}^{\circ}\right)\approx 5.67.$$

Equation (3) assumes symmetric deformation, quasi-static equilibrium, ideal joints, and negligible compliance in the frame and adhesive layer. In the fabricated device, bending of the PLA arms, joint flexibility, adhesive deformation, geometric imperfections, and viscoelastic relaxation may reduce the force transmitted to the PVDF film below the geometric upper bound. Across the free span, the film carries predominantly axial tension. At small deflections, bending is secondary, and the dominant stress is the in-plane normal stress $\sigma_{1}$ acting along the film length. This stress activates the $d_{31}$ mode, in which an in-plane stress along direction 1 produces an electric displacement along the through-thickness poling direction 3, as shown in Figure 1c.

Because the epoxy and PLA surfaces constrain the bonded ends, these regions undergo substantially less axial strain than the free span and contribute little to piezoelectric charge generation. The silver-ink electrodes nevertheless extend over the full film length, so the complete electrode-covered area contributes to the capacitance. This distinction is represented in Section 2.2 by the ratio (Equation (5))

(5) $$\frac{L_{\mathrm{mech},\mathrm{active}}}{L_{\mathrm{elec},\mathrm{active}}}.$$

The amplified tensile force defined in Equation (2) provides the mechanical input to the electromechanical model developed in the following subsection.

### 2.2. Theoretical Analysis

To predict the electrical response of the harvester from a known mechanical input, the analytical model is divided into two parts. The first describes the geometric force amplification of the flextensional frame, and the second relates the resulting PVDF tensile stress to the open-circuit voltage. Power delivered to an external resistive load is considered separately. The two parts are linked through the PVDF tensile force defined in Section 2.1.

#### 2.2.1. Mechanical Force-Amplification Model

In the fabricated device, bending of the PLA arms, joint flexibility, adhesive-layer deformation, manufacturing tolerances, and the viscoelastic behavior of PLA may reduce the force transmitted to the PVDF film below this ideal prediction [41,42].

A first-order equivalent-stiffness model is introduced to represent this reduction. The axial stiffness of the mechanically active PVDF span is

(6) $$k_{p}=\frac{E_{\mathrm{PVDF}}W_{\mathrm{PVDF},\mathrm{active}}t_{\mathrm{PVDF}}}{L_{\mathrm{mech},\mathrm{active}}},$$

where $E_{\mathrm{PVDF}}$, $W_{\mathrm{PVDF},\mathrm{active}}$, $t_{\mathrm{PVDF}}$, and $L_{\mathrm{mech},\mathrm{active}}$ denote the PVDF Young’s modulus, active width, thickness, and mechanically active length, respectively.

Under the small-displacement geometry, the PVDF elongation $u_{x}$ and the vertical platform displacement $u_{z}$ are related by $u_{x}=u_{z}\tan\theta$. The corresponding PVDF stiffness referring to the vertical loading direction is therefore

(7) $$k_{p,z}=\chi k_{p}\tan^{2}\theta ,$$

where χ is a dimensionless width-participation factor. Let $k_{f}$ denote the effective vertical stiffness associated with frame deformation that does not contribute directly to PVDF elongation. The force-transfer efficiency is then defined as the fraction of the input force carried through the PVDF load path (using Equations (6) and (7)):

(8) $$\eta_{F}=\frac{k_{p,z}}{k_{f}+k_{p,z}}={\left(1+\frac{k_{f}}{\chi k_{p}\tan^{2}\theta }\right)}^{-1}.$$

The effective tensile force transmitted to the PVDF film is consequently

(9) $$F_{\mathrm{PVDF},\mathrm{eff}}=\eta_{F}M_{F}F_{\mathrm{in}}.$$

In the ideal limit $k_{f}\to 0$, $\eta_{F}\to 1$, and Equation (9) reduces to the geometric upper-bound prediction. For $k_{f}>0$, the effective tensile force remains below this limit.

The arm angle therefore introduces a trade-off. Decreasing θ increases the geometric amplification $M_{F}=\cot\theta$ but decreases the referred PVDF stiffness because $k_{p,z}\propto \tan^{2}\theta$. A smaller angle consequently increases the sensitivity of the force transfer to frame deformation and joint flexibility. The value θ=10° was selected as a practical compromise between geometric amplification and structural stability over the tested load range.

The model applies only when the excitation frequency remains sufficiently below the first structural resonance of the assembled PLA–PVDF system. Near resonance, inertial forces, damping, and dynamic deformation become significant, and Equations (4)–(9) do not provide a complete description of the response. The off-resonance and resonance-dominated regions are identified through the impedance characterization presented in Section 4.1.

No local force or strain measurements were performed. Therefore, $\eta_{F}$ and the effective amplification factor $\eta_{F}M_{F}$ were not independently validated and were treated as model-based quantities rather than measured device properties.

#### 2.2.2. Electromechanical Model of the PVDF Film

Under force-controlled harmonic excitation, the applied input force is represented as

(10) $$F_{\mathrm{in}}\left(t\right)=F_{0}\sin\left(\omega t-\Delta \phi \right),\;\omega =2\pi f,$$

where $F_{0}$ is the peak force amplitude, $F_{0}=\sqrt{2}F_{\mathrm{in},\mathrm{rms}}$, ω is the angular frequency, and Δϕ is the phase of the force signal relative to the acquisition reference. Under quasi-static loading, the phase difference between the applied force and the intrinsic piezoelectric response is expected to be small.

Using the geometric force-amplification relation in Equation (3), the ideal tensile force in the PVDF film is

(11) $$F_{\mathrm{PVDF}}\left(t\right)=M_{F}F_{\mathrm{in}}\left(t\right).$$

Equation (11) represents the ideal geometric upper bound. The effective force in the fabricated device is reduced by the force-transfer efficiency $\eta_{F}$ defined in Equation (8).

The unelectroded overhang remained outside the frame and was excluded from the mechanically and electrically active regions. The mechanically active and electrode-covered areas are therefore

(12) $$A_{\mathrm{mech}}=L_{\mathrm{mech},\mathrm{active}}W_{\mathrm{PVDF},\mathrm{active}},$$

and

(13) $$A_{\mathrm{elec}}=L_{\mathrm{elec},\mathrm{active}}W_{\mathrm{PVDF},\mathrm{active}},$$

respectively. Here, $W_{\mathrm{PVDF},\mathrm{active}}$ is the common active width. The area $A_{\mathrm{mech}}$ (Equation (12)) represents the first-order charge-generating region, whereas $A_{\mathrm{elec}}$ (Equation (13)) determines the device capacitance.

Assuming uniform axial stress over the mechanically active span, the in-plane normal stress is

(14) $$\sigma_{1}\left(t\right)=\frac{F_{\mathrm{PVDF}}\left(t\right)}{W_{\mathrm{PVDF},\mathrm{active}}t_{\mathrm{PVDF}}},$$

where $t_{\mathrm{PVDF}}$ is the PVDF-layer thickness.

Within the piezoelectric charge-source representation, the source-charge density generated in the $d_{31}$ mode is

(15) $$D_{3,p}\left(t\right)=d_{31}\sigma_{1}\left(t\right),$$

where $D_{3,p}$ denotes the piezoelectrically generated component of the electric displacement, and $d_{31}$ is the transverse piezoelectric coefficient [25,47]. The source charge generated over the mechanically active electrode area, using Equation (15), is

(16) $$Q_{p}\left(t\right)=D_{3,p}\left(t\right)A_{\mathrm{mech}}=d_{31}\sigma_{1}\left(t\right)L_{\mathrm{mech},\mathrm{active}}W_{\mathrm{PVDF},\mathrm{active}}.$$

Substituting Equations (11) and (14) into Equation (16) gives

(17) $$Q_{p}\left(t\right)=d_{31}\frac{L_{\mathrm{mech},\mathrm{active}}}{t_{\mathrm{PVDF}}}M_{F}F_{\mathrm{in}}\left(t\right).$$

The intrinsic capacitance of the electrode-covered PVDF region is

(18) $$C_{P}\equiv C_{\mathrm{PVDF}}=\frac{\varepsilon_{33}^{T}L_{\mathrm{elec},\mathrm{active}}W_{\mathrm{PVDF},\mathrm{active}}}{t_{\mathrm{PVDF}}},$$

where $\varepsilon_{33}^{T}$ is the constant-stress permittivity. The full electrically active length is used because the bonded end regions remain electrically connected and contribute to the total capacitance.

The nominal values $d_{31}=-25\;\mathrm{pC}/N$ and $\varepsilon_{33}^{T}=1.14\times 10^{-10}\;F/m$ were obtained from the manufacturer specification for the commercially poled 100μm PVDF film [48]. The film is poled through its thickness, corresponding to direction 3, while the intended tensile loading direction is direction 1. The effective $d_{31}$ coefficient of the bonded film was not measured independently; therefore, the manufacturer value was used only for the nominal analytical prediction.

For numerical comparison with the assembled prototype, the measured capacitance $C_{P}\approx 12.5\;\mathrm{nF}$ was used directly rather than assuming that the manufacturer permittivity exactly reproduced the capacitance of the bonded device.

The PVDF film is represented by a piezoelectric charge source in parallel with its capacitance. Under ideal open-circuit conditions,

(19) $$V_{\mathrm{oc},\mathrm{ideal}}\left(t\right)=\frac{Q_{p}\left(t\right)}{C_{P}}.$$

Substituting Equations (17) and (18) into Equation (19) gives

(20) $$\begin{array}{cc} V_{\mathrm{oc},\mathrm{ideal}}\left(t\right) & =\frac{d_{31}L_{\mathrm{mech},\mathrm{active}}}{t_{\mathrm{PVDF}}C_{P}}M_{F}F_{\mathrm{in}}\left(t\right)\\ \; & =\frac{d_{31}M_{F}}{\varepsilon_{33}^{T}W_{\mathrm{PVDF},\mathrm{active}}}\left(\frac{L_{\mathrm{mech},\mathrm{active}}}{L_{\mathrm{elec},\mathrm{active}}}\right)F_{\mathrm{in}}\left(t\right). \end{array}$$

Equation (20) is the ideal upper-bound voltage because it assumes that the full geometric amplification is transmitted to the PVDF film. Using the force-transfer efficiency derived in Equation (8), the effective model becomes

(21) $$\begin{array}{cc} V_{\mathrm{oc}}\left(t\right) & =\eta_{F}V_{\mathrm{oc},\mathrm{ideal}}\left(t\right)\\ \; & =\eta_{F}\frac{d_{31}M_{F}}{\varepsilon_{33}^{T}W_{\mathrm{PVDF},\mathrm{active}}}\left(\frac{L_{\mathrm{mech},\mathrm{active}}}{L_{\mathrm{elec},\mathrm{active}}}\right)F_{\mathrm{in}}\left(t\right),\;0<\eta_{F}\leq 1. \end{array}$$

The parameter $\eta_{F}$ accounts only for the mechanical reduction in force transfer. It was not measured independently and was therefore retained as a model-based parameter. Uncertainty in the effective piezoelectric coefficient caused by the in-plane orientation of the uniaxially drawn film was treated separately and was not included in $\eta_{F}$. The manufacturer reports that the transverse in-plane coefficient $d_{32}$ is substantially smaller than $d_{31}$ [48]; therefore, misalignment between the film draw direction and the loading direction may reduce the effective response.

The finite input resistance of the measurement instrument attenuates the measured voltage relative to the ideal open-circuit value. For a continuously driven piezoelectric charge source in parallel with $C_{P}$ and loaded by the input resistance $R_{\mathrm{in}}$, the appropriate steady-state voltage transfer ratio is [49]

(22) $$\left|H(\omega )\right|=\frac{\left|V_{\mathrm{meas}}\right|}{\left|V_{\mathrm{oc}}\right|}=\frac{\omega R_{\mathrm{in}}C_{P}}{\sqrt{1+{\left(\omega R_{\mathrm{in}}C_{P}\right)}^{2}}}.$$

Equation (22), rather than a single-discharge half-cycle model, is used to evaluate the frequency-dependent measurement attenuation.

The instantaneous electrostatic energy stored in the effective PVDF capacitance is

(23) $$E_{C}\left(t\right)=\frac{1}{2}C_{P}{\left[V(t)\right]}^{2}.$$

This quantity is the energy temporarily stored in the PVDF capacitance at voltage V(t); it is not the total generated, delivered, or harvested electrical energy.

When the generated voltage is measured across an external resistive load $R_{L}$, the instantaneous power delivered to the load is

(24) $$P_{L}\left(t\right)=\frac{{\left[V_{L}\left(t\right)\right]}^{2}}{R_{L}}.$$

The average power delivered to the resistive load over one excitation period *T* is

(25) $$\begin{array}{cc} P_{\mathrm{avg}} & =\frac{1}{T}\int_{0}^{T}\frac{{\left[V_{L}\left(t\right)\right]}^{2}}{R_{L}}\;dt\\ \; & =\frac{V_{\mathrm{rms}}^{2}}{R_{L}}=\frac{V_{\mathrm{peak}}^{2}}{2R_{L}}, \end{array}$$

where the final equality applies to a sinusoidal voltage across the resistive load.

## 3. Device Prototype Fabrication and Characterization

### 3.1. Prototype Fabrication

A commercially poled single-layer PVDF film with screen-printed silver-ink electrodes was used as the piezoelectric transduction element. The nominal PVDF-layer thickness was $t_{\mathrm{PVDF}}=100\;\mu m$, excluding the electrode layers. The electrode-covered region had a length $L_{\mathrm{PVDF}}=113.3\;\mathrm{mm}$ and an active width $W_{\mathrm{PVDF},\mathrm{active}}=76.0\;\mathrm{mm}$ [48].

The nominal silver-ink electrode thickness was approximately $t_{\mathrm{Ag}}=6\;\mu m$ on each face. The sheet also contained an unelectroded PVDF overhang approximately 10mm wide and extending along the 113.3mm film length. This overhang remained outside the PLA frame and was not bonded to the structure. It was therefore excluded from the mechanically active area, electrically active area, capacitance calculation, and first-order axial-stiffness model. Accordingly, the width-participation factor used in the present model is χ=1.

The PVDF film was bonded to the PLA frame only at its two end regions, each with a bonded length of $L_{\mathrm{glue}}=12.2\;\mathrm{mm}$. The mechanically active length, defined as the unbonded central span that undergoes axial deformation, was

(26) $$\begin{array}{cc} L_{\mathrm{mech},\mathrm{active}} & =L_{\mathrm{PVDF}}-2L_{\mathrm{glue}}\\ \; & =113.3-2(12.2)=88.9\;\mathrm{mm}. \end{array}$$

The bonded end regions provided the axial constraint required to transfer force into the PVDF film and defined the boundaries of the mechanically active span (Equation (26)). The electrically active length, $L_{\mathrm{elec},\mathrm{active}}=113.3\;\mathrm{mm}$, corresponded to the full electrode-covered length because the silver-ink electrodes extended continuously across the active region. The active width was

(27) $$W_{\mathrm{active}}=W_{\mathrm{PVDF},\mathrm{active}}=76.0\;\mathrm{mm}.$$

The distinction between $L_{\mathrm{mech},\mathrm{active}}$ and $L_{\mathrm{elec},\mathrm{active}}$ (with the active width defined in Equation (27)) is discussed in Section 2.1 and Section 2.2. Figure 1c identifies the bonded, mechanically active, and electrically active regions.

The flextensional frame was fabricated from PLA by fused-deposition modeling (FDM) using 100% infill density and a layer height of 0.20mm. The high infill density was selected to reduce porosity-induced compliance, while the selected layer height provided a practical balance between dimensional accuracy and fabrication time. The frame was printed with its primary load-bearing features aligned with the in-plane print direction.

The geometric, material, and fabrication parameters of the prototype are summarized in Table 1.

FDM-printed PLA at 100% infill typically exhibits a Young’s modulus of approximately 2.5–3.5GPa along the print direction [41,42]. The flextensional motion of the present frame, however, involves arm bending, junction rotation, local deformation, and axial extension. Therefore, an axial-stiffness calculation for an idealized straight arm would not provide a reliable estimate of the effective force transmitted to the PVDF film. Determining the effective frame stiffness would require the complete CAD geometry, appropriate junction boundary conditions, and a coupled structural or finite-element model.

The axial stiffness of the mechanically active PVDF span, excluding the silver-ink electrode layers, is

(28) $$\begin{array}{cc} k_{p}\equiv k_{\mathrm{PVDF}} & =\frac{E_{\mathrm{PVDF}}W_{\mathrm{PVDF},\mathrm{active}}t_{\mathrm{PVDF}}}{L_{\mathrm{mech},\mathrm{active}}}\\ \; & \approx \frac{\left(2.5\times 10^{3}\;N/{\mathrm{mm}}^{2}\right)\left(76.0\;\mathrm{mm}\right)\left(0.100\;\mathrm{mm}\right)}{88.9\;\mathrm{mm}}\\ \; & \approx 213.7\;N/\mathrm{mm}. \end{array}$$

The electrode layers were excluded from Equation (28) because their effective axial properties in the bonded film were not independently characterized. Their possible contribution to the composite stiffness remains a source of modeling uncertainty. The force-transfer efficiency $\eta_{F}$ introduced in Equation (8) was not measured independently, and no numerical value was assigned using a simplified arm-stiffness calculation.

Before bonding, the PLA contact surfaces were cleaned and lightly abraded to improve adhesive wetting and mechanical interlocking. A thin layer of J-B Weld ClearWeld Syringe Epoxy Adhesive (J-B Weld, Sulphur Springs, TX, USA) was applied within the two bonded end regions.

During bonding, the PVDF film was held only sufficiently taut to remove visible slack and maintain a flat, wrinkle-free configuration. No prescribed or controlled pretensile force or prestrain was applied, and any residual tension introduced during manual assembly was not measured. The adhesive layer was maintained below 100μm to limit compliance in the force-transfer path. The assembly was clamped and cured at room temperature for 24 h before mechanical or electrical testing.

### 3.2. Experimental Setup and Measurement Procedure

**Figure 3:**
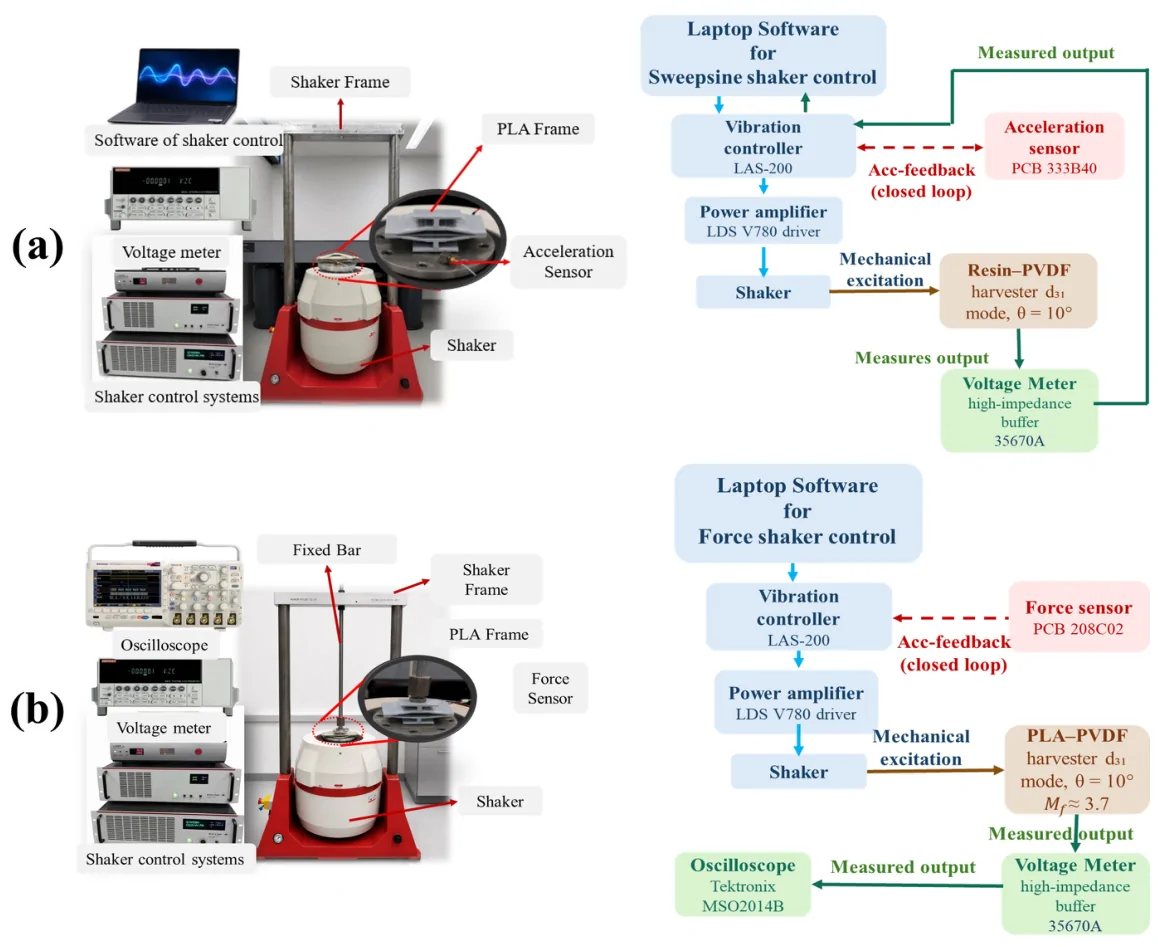
Two experimental measurements methods used for energy harvesting characterizations (**a**) Force-controlled testing for off-resonance mode operation. (**b**) Acceleration-controlled testing for resonance mode operation. (The arrows in the logical diagrams are the signal flow directions).

Dynamic characterization of the FPPFEH was performed using an electrodynamic shaker (LDS V780, Brüel & Kjær LDS, Nærum, Denmark) driven by a power amplifier and controlled using an LAS-200 Laser USB Shaker Control System. Two experimental configurations were employed, as shown in Figure 3: a force-controlled configuration for off-resonance testing and an acceleration-controlled configuration for sweep-sine and resonance testing.

For force-controlled characterization, a rigid fixed-bar fixture was installed above the shaker, as shown in Figure 3a. The upper loading platform of the FPPFEH was connected to the fixed structure through a rigid loading rod and an inline force sensor. Relative motion between the shaker table and the fixed bar generated a controlled dynamic compressive force on the harvester. The loading rod was aligned vertically with the center of the upper platform to minimize off-axis loading and torsional moments and to promote symmetric loading of the inclined frame arms, consistent with the assumptions of the force-amplification model described in Section 2.1.

A calibrated inline ICP quartz force sensor (PCB Piezotronics, Depew, NY, USA, Model 208C02; manufacturer-specified sensitivity tolerance of ±0.5%) was installed between the loading rod and the upper platform to measure the applied dynamic force $F_{\mathrm{in}}$. The measured force signal was used as closed-loop feedback, enabling repeatable harmonic excitation at prescribed RMS force levels. This configuration was used for the off-resonance force-controlled experiments reported in Section 4.2, Section 4.3 and Section 4.4.

Because the Model 208C02 is an ICP quartz force sensor, its output is AC-coupled and represents only the time-varying component of the applied load. The static preload established when the loading rod was brought into contact with the upper platform was not recorded. Consequently, all reported force levels are dynamic RMS values about an unmeasured mean force. This limitation does not prevent comparison of the dynamic force and voltage amplitudes, but it prevents determination of the absolute mean stress and the minimum and maximum PVDF tensile forces during each loading cycle.

For acceleration-controlled sweep-sine and resonance testing, the fixed-bar fixture, loading rod, and inline force sensor were removed. The harvester was mounted directly on the shaker table without an external upper constraint, as shown in Figure 3b. The shaker then provided base excitation, and the response was characterized as a function of excitation frequency and acceleration level. A miniature accelerometer (PCB Piezotronics, Model 333B40) mounted on the shaker table was used to monitor and control the applied base acceleration. This configuration was used for the measurements reported in Section 4.5 and Section 4.6.

The terminal voltage $V_{AB}$ was measured using an Agilent 35670A dynamic signal analyzer with a nominal input resistance of 1MΩ and recorded using a digital oscilloscope (Tektronix MSO2014B, Tektronix Inc., Beaverton, OR, USA). Because the measurement resistance was finite, the recorded voltage did not represent an ideal open-circuit condition over the complete frequency range.

For the energy-storage experiments, the FPPFEH output was connected to a full-bridge silicon rectifier followed by a 6600μF supercapacitor (Model EHC-601, Piezo Systems Inc., Cambridge, MA, USA). The rectified voltage $V_{CD}$ across the supercapacitor was recorded continuously during charging to evaluate the practical energy-storage response of the harvester [43].

Electrical impedance characterization was performed separately using a precision impedance analyzer (Agilent 4294A, Keysight Technologies, Santa Rosa, CA, USA) under small-signal sinusoidal electrical excitation. The measurements were conducted with the PVDF film bonded within the PLA frame and without external shaker excitation. The assembled device remained in its fabricated configuration during these measurements.

The impedance measurements provided the capacitance, dielectric-loss, resonance, and anti-resonance characteristics of the assembled transducer. Because the nominal lower-frequency limit of the Agilent 4294A is 40 Hz, capacitance below 40 Hz was not measured directly using this instrument. The approximately constant capacitance measured over the off-resonance 40–50 Hz interval was used as representative of the broader low-frequency operating region. The impedance results were used to distinguish the quasi-static off-resonance regime from the resonance-dominated regime before the acceleration-controlled shaker tests.

(29) $$\frac{1.45\times 10^{-10}}{1.14\times 10^{-10}}\approx 1.27.$$

(30) $$E_{\mathrm{stored}}=\frac{1}{2}C_{\mathrm{SC}}V_{\mathrm{SC}}^{2}=\frac{1}{2}\left(6600\times 10^{-6}\right){\left(2.10\right)}^{2}\approx 14.55\;\mathrm{mJ},$$

(31) $$\frac{C_{\mathrm{SC}}}{C_{\mathrm{PVDF}}}=\frac{6600\times 10^{-6}}{12.5\times 10^{-9}}\approx 528,000.$$

(32) $$V_{\mathrm{oc}}\left(f\right)=V_{\mathrm{meas}}\left(f\right)\frac{\sqrt{1+{\left(\omega R_{\mathrm{in}}C_{P}\right)}^{2}}}{\omega R_{\mathrm{in}}C_{P}}.$$

(33) $$R_{\mathrm{opt}}=\frac{1}{2\pi f_{\mathrm{peak}}C_{P}}=\frac{1}{2\pi \times 114\times 12.5\times 10^{-9}}\approx 111\;k\Omega .$$

## 4. Results and Discussion

### 4.1. Capacitance and Dielectric Loss Characterization

**Figure 4:**
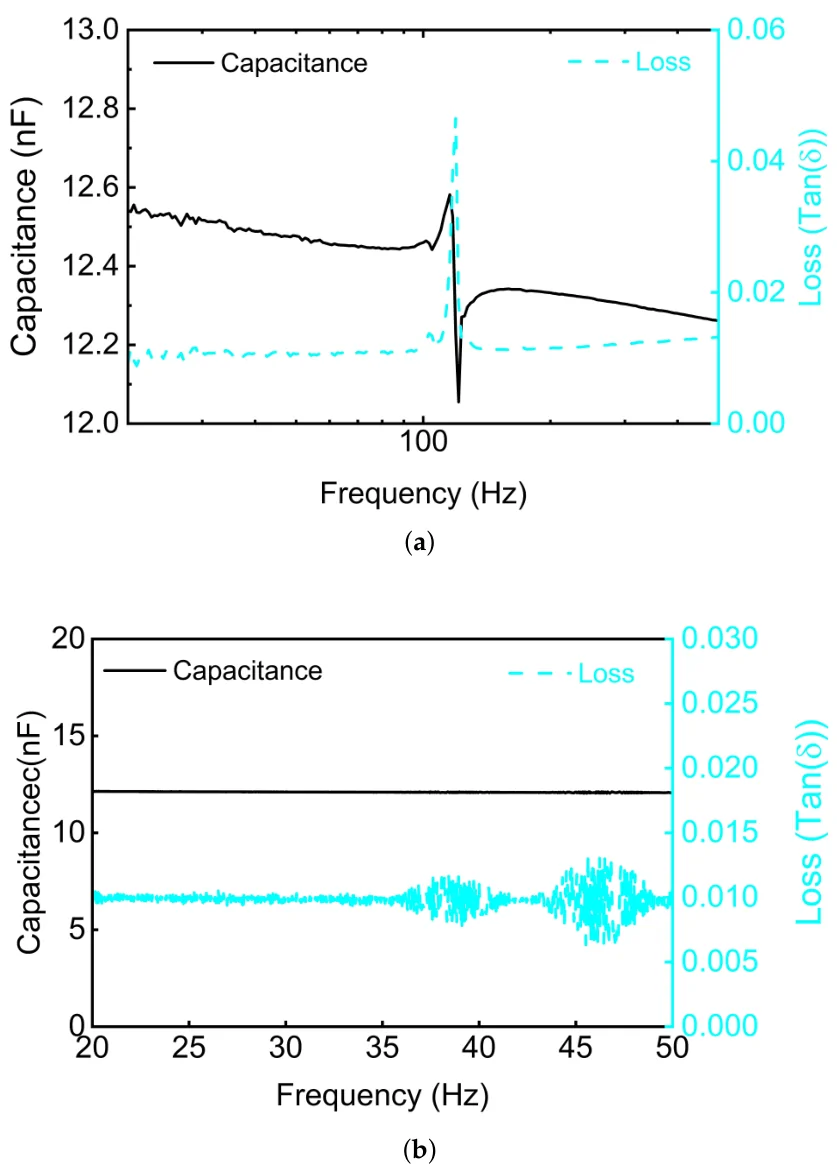
Measured capacitance $C_{P}$ and dielectric loss factor tanδ of the FPPFEH. (**a**) Frequency response showing a resonance-associated loss peak near 100–105 Hz. (**b**) Enlarged view of the off-resonance region (20–50 Hz), demonstrating stable capacitance and low dielectric loss.

Frequency-dependent electrical characterization was performed to evaluate the dielectric response of the PVDF layer following integration with the PLA flextensional frame. Figure 4 shows the measured capacitance $C_{P}$ and dielectric loss factor tanδ of the clamped PVDF layer as a function of frequency. As shown in Figure 4a, the capacitance exhibits a resonance near 104 Hz and an anti-resonance near 114 Hz. In the off-resonance region (20–50 Hz), shown in Figure 4b, the capacitance remains nearly constant at approximately 12.5 nF and the dielectric loss stays low and stable throughout, indicating minimal energy dissipation within the film at these frequencies. This flat, low-loss behavior confirms that the electrical response in this frequency band is dominated by the intrinsic dielectric properties of the PVDF rather than by any frequency-dependent structural or interfacial effects.

To verify that bonding the film to the PLA frame did not meaningfully alter its intrinsic electrical properties, the clamped response was compared against measurements taken from the free PVDF sheet prior to assembly. The free film exhibited a nearly constant capacitance of approximately 12.0 nF across the same frequency range. After integration with the PLA frame, the capacitance increased slightly to 12.4–12.6 nF, a change of less than 5%. This small upward shift might come from the slight deformation of the PVDF film due to a small extension in the length of the film. The close agreement between the free and clamped responses confirmed that the dielectric characteristics of the PVDF layer were largely preserved after structural integration and that the value $C_{P}\approx 12.5$ nF used throughout the electromechanical model was representative of the assembled device.

The measured capacitance also provides a direct check on the permittivity assumed in the electromechanical model. Substituting $C_{P}=12.5$ nF and the electrode geometry reported in Table 1 into Equation (18) gives $\varepsilon_{33}^{T}=1.45\times 10^{-10}\;F/m$ ($\varepsilon_{r}\approx 16.4$), compared with the manufacturer-reported value of $\varepsilon_{33}^{T}=1.14\times 10^{-10}\;F/m$ ($\varepsilon_{r}\approx 12.9$). This difference is consistent with the frequencies at which the two values apply. Manufacturer permittivity values for PVDF are conventionally reported at 1 kHz, whereas the present device operates at 20–50 Hz, where low-frequency dielectric relaxation can increase $\varepsilon_{33}^{T}$. Because the open-circuit voltage in Equation (20) scales inversely with permittivity, $V_{\mathrm{oc}}\propto 1/\varepsilon_{33}^{T}$, using the 1 kHz manufacturer value would overpredict $V_{\mathrm{oc}}$ by the factor given in Equation (29). Therefore, the permittivity obtained from the measured capacitance was used throughout the analysis, and the corresponding correction factor is explicitly included in the loss budget presented in Section 4.2.

Based on these observations, two distinct operating regimes can be identified for the PLA–PVDF harvester, as summarized in Table 2.

### 4.2. Off-Resonance Validation Under Constant-Frequency Excitation

To validate the quasi-static electromechanical model developed in Section 2.2, force-controlled excitation experiments were performed under off-resonance conditions at a fixed frequency of 30 Hz. This frequency lies well below the structural resonance at 104 Hz and the anti-resonance at 114 Hz, placing the device firmly within the quasi-static operating regime where the measured capacitance is stable and the electrical response is governed by mechanically induced charge generation rather than frequency-dependent dynamic effects. At 30 Hz, the attenuation of the measured voltage due to the finite input impedance, evaluated from Equation (22), is approximately 8% and is corrected where the open-circuit voltage is required.

Force amplitudes were varied across five levels spanning 3.52–19.12 N_rms_ while the excitation frequency was held constant. According to the quasi-static formulation of Equation (20), the open-circuit voltage should scale linearly with the applied force at a fixed frequency. The measured peak open-circuit voltage at each level was compared against the ideal analytical prediction of Equation (20), and the results are summarized in Table 3. The peak force used in the model was computed as $F_{0}=\sqrt{2}F_{\mathrm{in},\mathrm{rms}}$, consistent with the sinusoidal force waveform applied by the shaker system.

The ideal model of Equation (21), evaluated with the datasheet coefficient $d_{31}=25\;\mathrm{pC}/N$ and $\eta_{F}=1$, overpredicts the measured voltage at every force level. Crucially, the ratio $V_{\mathrm{model}}/V_{\exp}$ is nearly constant across the three intermediate force levels (7.4–7.5 while the applied force nearly doubles) and rises only at the extremes. Two mechanisms account for this constant offset, both introduced in Section 2.2. First, the elastic compliance of the polymer frame reduces the force actually delivered to the film below the geometric upper bound, represented by the force-transfer efficiency $\eta_{F}$ of Equation (8); the arm bending stiffness estimated in Section 3 places $\eta_{F}$ in the range 0.2–0.8. Second, the uniaxially oriented PVDF film is strongly anisotropic in plane, with a transverse coefficient $d_{32}$ substantially smaller than $d_{31}$, so the active coefficient depends on the film draw-axis orientation relative to the loading axis. Neither quantity was measured independently in this work; taken together, they are sufficient to account for the observed offset, and their separate determination is identified as future work.

The larger deviations at the lowest force (3.52 N_rms_) and highest force (19.12 N_rms_) are superimposed load-dependent effects. At low force, residual slack or contact compliance in the epoxy bond line prevents full axial load transfer below a threshold. At high force, the film may fall out of tension during part of the cycle, since a thin film supports tension only; the static preload was not recorded because the inline force sensor is AC-coupled, so the minimum instantaneous force per cycle is unknown. Both effects reduce the measured voltage relative to the constant-offset trend.

**Figure 5:**
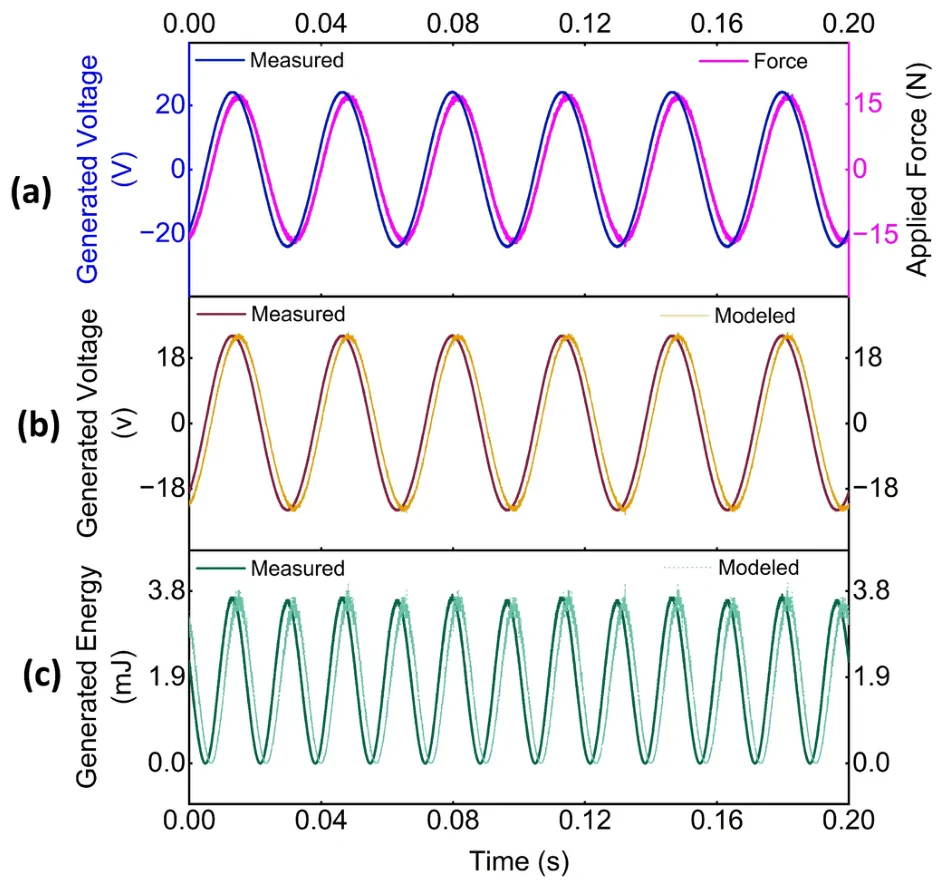
Time-domain response of the FPPFEH at 12.32 N_rms_ and 30 Hz. (**a**) Measured open-circuit voltage and applied force, showing that the voltage tracks the force with a small phase lag. (**b**) Measured generated voltage. (**c**) Measured generated energy, oscillating at twice the excitation frequency.

The time-domain response at 12.32 N_rms_ is shown in Figure 5.

The measured voltage waveform follows the applied sinusoidal force, as shown in Figure 5a, with stable periodic behavior and a small phase offset, consistent with the harmonic expression in Equation (10). From an energy perspective, the cycle-level generated electrical energy shown in Figure 5c oscillates at twice the excitation frequency, as expected from the squared voltage dependence in Equation (23). The quadratic scaling of both energy and power with voltage, described by Equations (23)–(25), has an important practical implication: any error in voltage prediction is squared when translated into energy or power.

### 4.3. Rectified Energy Storage Performance at 30 Hz

The feasibility of energy storage capability of the harvester was evaluated under harmonic excitation at f=30 Hz with an input force of 12.32 N_rms_. This operating condition lies well below the structural resonance identified in Section 4.1 and therefore represents the off-resonant quasi-static regime in which the force-amplification model is best validated. The harvester output was connected to a full-bridge silicon rectifier followed by a 6,600 μF supercapacitor to assess the feasibility of converting low-frequency mechanical excitation into stored electrical energy under realistic circuit conditions.

**Figure 6:**
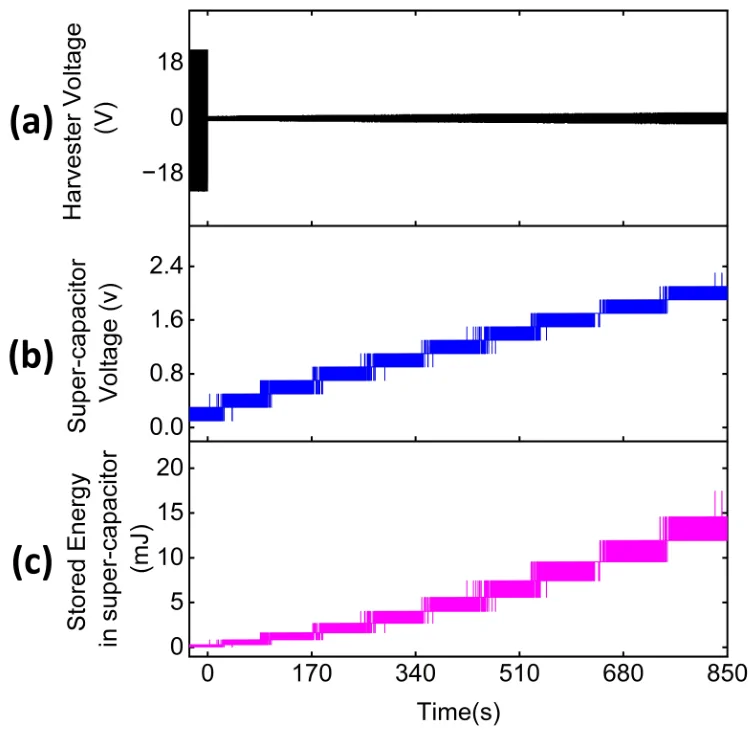
A 6600 μF supercapacitor charged by the FPPFEH under 12.32 N_rms_ dynamic force at 30 Hz. (**a**) Generated AC voltage of the FPPFEH before and during the charging process. (**b**) Voltage of the charged supercapacitor during the process. (**c**) Stored electrical energy in the supercapacitor.

Figure 6 shows the generated AC voltage waveform, the rectified supercapacitor terminal voltage, and the corresponding accumulated stored energy as functions of time.

The generated voltage exhibits a stable, symmetric periodic waveform before charging the supercapacitor. After rectification, the charged supercapacitor voltage increases in a stepwise fashion with each successive vibration cycle, reflecting the discrete charge packets transferred per half-cycle through the bridge rectifier. This incremental charging profile is consistent with the energy-transfer mechanism described by Zhao et al. [43] for piezoelectric harvesters coupled to capacitive storage elements, where the charge transferred per cycle is governed by the ratio of the source capacitance to the storage capacitance.

Under the applied operating conditions, the supercapacitor terminal voltage reaches approximately 2.10 V after 14 min of continuous excitation. The energy stored in the supercapacitor at this point is calculated directly using Equation (30) [43], where $C_{\mathrm{SC}}=6600\;\mu F$ is the supercapacitor capacitance and $V_{\mathrm{SC}}=2.10\;V$ is the measured terminal voltage at the end of the charging period.

Several practical factors limit the charging efficiency of this configuration and are worth quantifying explicitly. First, the full-bridge silicon rectifier introduces a total forward voltage drop of approximately 2×0.7V=1.4V across the two conducting diodes at any instant. This threshold means that the supercapacitor begins charging only once the PVDF open-circuit voltage exceeds approximately 1.4 V per half-cycle, and the effective charging voltage available to the supercapacitor is reduced by this amount throughout the process. At the tested condition, the peak open-circuit voltage is approximately 24.4 V (Table 3), so the diode drop represents less than 6% of the peak voltage and its effect on the final stored energy is modest. However, at lower force levels where the peak voltage approaches the diode threshold, this loss would become increasingly significant.

Second, and more fundamentally, the capacitance ratio between the supercapacitor and the PVDF source is given by Equation (31) [43].

This extreme mismatch means that each vibration cycle transfers only a tiny fraction of the charge stored in the PVDF capacitance to the supercapacitor, which explains the slow stepwise charging profile visible in Figure 6. The supercapacitor voltage rises by only a small increment per cycle, and a very large number of cycles, approximately 30×840=25,200 cycles over the 14 min test, are required to accumulate the observed terminal voltage of 2.10 V.

The monotonic growth of the supercapacitor voltage throughout the 14 min test confirms that the PLA flextensional frame reliably converts low-frequency mechanical excitation into usable electrical energy under realistic off-resonant conditions.

### 4.4. Frequency–Voltage Response Under Off-Resonance Excitation

To further characterize the electromechanical behavior within the off-resonance regime, the open-circuit voltage was measured across the frequency range of 10–50 Hz at five discrete force-amplitude levels spanning 1–6 N_rms_. These measurements complement the single-frequency, multi-force validation of Section 4.2 by confirming that the quasi-static voltage response is stable across the full intended operating bandwidth, not just at the 30 Hz validation point.

**Figure 7:**
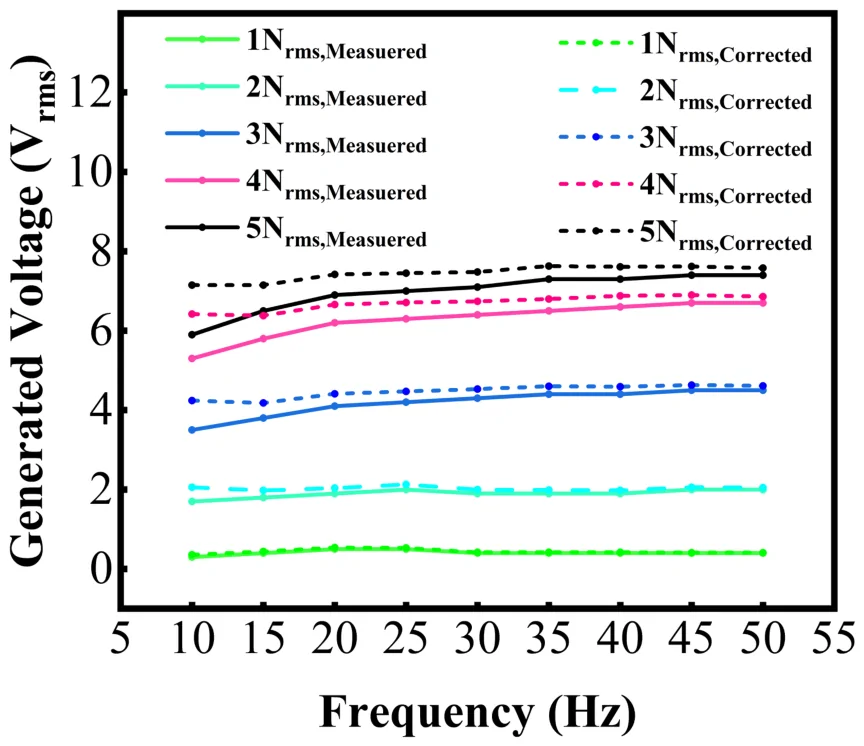
Measured open-circuit voltage as a function of excitation frequency from 10 to 50 Hz for applied force amplitudes of 1–6 N_rms_.

Figure 7 presents the measured voltage–frequency response at each force level. Across the investigated band, the generated voltage remains relatively stable with only minor variation, indicating that the electrical output is nearly frequency-independent within the 10–50 Hz off-resonance region. This behavior is the expected signature of quasi-static electromechanical operation: according to Equation (20), the open-circuit voltage depends on the applied force amplitude and the geometric coupling coefficient Γ, but not directly on frequency. At any fixed excitation frequency, the generated voltage increases systematically with increasing force amplitude, consistent with the linear force–voltage relation in Equation (20). According to Equations (24) and (25), the generated electrical power scales as the square of the applied force. Ideally, the voltage at a fixed force level should be constant across the tested frequency band, since Equation (21) contains no explicit frequency dependence. In practice, a slight downward deviation from this flat response is observed at the lower end of the range, particularly below 20 Hz. This deviation does not originate in the device; it is an artifact of the finite input impedance of the measurement instrument, which together with the source capacitance $C_{P}$ forms a first-order high-pass network.

Because the PVDF film is driven continuously by the shaker rather than discharged from a fixed initial charge, the appropriate description is the steady-state response of a sinusoidal charge source loaded by the instrument, not a single-shot RC discharge. Modeling the transducer as a charge source in parallel with $C_{P}$ and the instrument input resistance $R_{\mathrm{in}}$, the ratio of the measured voltage to the true open-circuit voltage is given by Equation (22) [49].

This ratio is a monotonically increasing high-pass function of frequency, bounded above by unity. The associated RC time constant and cutoff frequency are $\tau =R_{\mathrm{in}}C_{P}$ and $f_{c}=1/\left(2\pi R_{\mathrm{in}}C_{P}\right)$, respectively. Since $f_{c}\approx 12.7\;\mathrm{Hz}$ lies within the lower part of the tested band, Equation (22) predicts a measurable attenuation only at the lowest frequencies: approximately 38% at 10 Hz, 16% at 20 Hz, 8% at 30 Hz, and 3% at 50 Hz. To confirm that this attenuation, rather than any intrinsic frequency dependence, was responsible for the observed roll-off, the true open-circuit response was reconstructed by dividing each measured value by the transfer function of Equation (22), as expressed in Equation (32).

The reconstructed curves are shown as dashed lines in Figure 7. The effective time constant used in the reconstruction, $R_{\mathrm{in}}C_{P}\approx 19.6\;\mathrm{ms}$, was obtained directly from the measured data as the value that rendered the reconstructed response flat; with $C_{P}=12.5\;\mathrm{nF}$, this corresponds to an effective input impedance of approximately 1.57MΩ, consistent with the combined loading of the oscilloscope, the high-impedance buffer, and the connecting cabling rather than the nominal 1MΩ oscilloscope input alone. After this correction, the reconstructed voltage is flat to within about 1% across the entire 10–50 Hz band for all force levels.

### 4.5. Resonance Mode Operation

**Figure 8:**
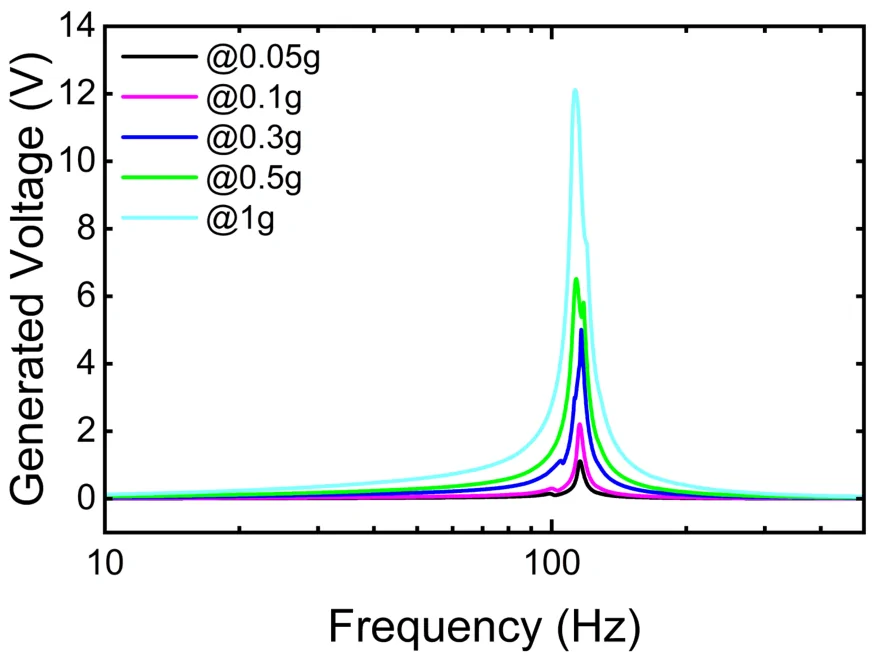
Measured open-circuit peak voltage versus excitation frequency for multiple base acceleration levels (0.05–1 g).

Figure 8 shows the measured open-circuit peak voltage as a function of excitation frequency for base acceleration levels ranging from 0.05 g to 1 g. A pronounced voltage amplification appears in the 112–116 Hz range across all tested acceleration levels, identifying this band as the first structural resonance of the FPPFEH. The resonance peak values extracted from Figure 8 are summarized in Table 4. The resonance frequency remains within a narrow interval of 112.88–116.49 Hz across the full tested acceleration range, a total variation of 3.2%. Above 0.3 g, however, the peak frequency falls monotonically from 116.49 Hz to 112.88 Hz, a shift of 3.6 Hz. A downward shift of the resonance frequency with increasing drive amplitude is the characteristic signature of stiffness softening and is consistent with amplitude-dependent behavior of the polymer frame and the adhesive bond line. The peak voltage increases with acceleration level, reaching 12.11 V at 1 g. Normalized by acceleration, however, the sensitivity $V_{\mathrm{peak}}/g$ falls from 22.2 V/g at 0.05 g to 12.1 V/g at 1 g. Extrapolating linearly from the lowest tested acceleration, 1 g would be expected to produce 22.2 V; the measured 12.11 V represents a shortfall of 45%. The departure from linearity is therefore substantial rather than marginal and grows monotonically with drive level: 9% at 0.1 g, 25% at 0.3 g, and 41% at 0.5 g. Several mechanisms may contribute to this behavior. First, at high vibration levels, the PVDF film may be stretched during only half of each vibration cycle because of its limited pre-stress. Second, amplitude-dependent damping in the PLA frame and at the adhesive interface may act together with the mild stiffness softening evident in the peak frequency. Within the resonance regime, the device therefore exhibits a genuinely nonlinear response, and output at high drive levels cannot be extrapolated from low-level measurements. This does not affect the quasi-static model validated in Section 4.2, Section 4.3 and Section 4.4, which applies well below resonance.

To quantify the sharpness of the resonance and the degree of dynamic amplification, the −3 dB bandwidth Δf and quality factor *Q* were extracted from the voltage–frequency response in Figure 8. The bandwidth is defined as the frequency interval over which the voltage falls to $1/\sqrt{2}$ of its peak value, and the quality factor follows as $Q=f_{\mathrm{peak}}/\Delta f$. The extracted values are listed in Table 4.

The observed voltage amplification near resonance is physically consistent with the electromechanical model of Equation (21), interpreted in the context of dynamic loading. Under harmonic excitation near resonance, the structural dynamics of the FPPFEH produce inertial amplification of the effective axial force applied to the PVDF film. The actual dynamic force in the film exceeds the quasi-static value $M_{F}F_{\mathrm{in}}$ because the frame is vibrating near its natural frequency. This dynamic force amplification is superimposed on the geometric force amplification of the flextensional mechanism, producing the sharp voltage peak observed in Figure 8. The voltage amplification near resonance therefore has two contributing sources: the geometric leverage of the frame, captured by $M_{F}=5.67$, and the dynamic magnification of the structural response, characterized by the *Q*-factor.

The resonance frequency identified in the shaker-based voltage response (112–116 Hz) lies approximately 8–12 Hz above the resonance identified through impedance characterization in Section 4.1 (104 Hz) and coincides with the anti-resonance frequency (114 Hz). A piezoelectric transducer resonates at the resonance frequency $f_{r}$ under short-circuit electrical boundary conditions and at the anti-resonance frequency $f_{a}$ under open-circuit conditions. The impedance sweep, which excites the transducer electrically, resolves both features, whereas the shaker measurement records open-circuit voltage through a high-impedance input and must therefore peak at $f_{a}$. The observed 112–116 Hz band brackets $f_{a}=114$ Hz. The agreement between the two independent measurements under their respective boundary conditions confirms the identification of the 104 Hz and 114 Hz features as the resonance and anti-resonance of the assembled device.

### 4.6. Power Delivery on Resistive Loads

The load-dependent electrical response of the harvester was evaluated under a fixed base acceleration of 0.1 g to quantify voltage generation and power extraction across a range of resistive loading conditions. The excitation frequency was swept from 80 to 140 Hz, covering the structural resonance band identified in Section 4.1 and Section 4.5. Four discrete load resistances were tested: 100, 150, 200, and 250 kΩ.

**Figure 9:**
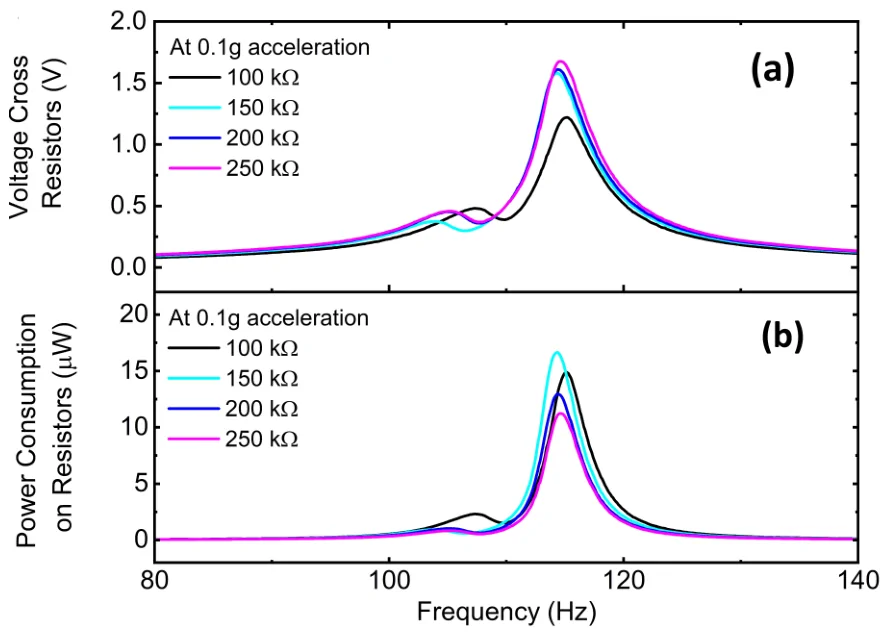
Measured (**a**) voltage magnitude and (**b**) electrical power across different resistive loads under 0.1 g base excitation.

Figure 9 presents the measured terminal voltage magnitude and the corresponding electrical power delivered to each resistive load as functions of frequency. As expected, the terminal voltage increases monotonically with load resistance across the full frequency range: as $R_{\mathrm{Load}}$ increases, less current is drawn from the PVDF source, and the terminal voltage approaches the open-circuit limit. The voltage curves for all four resistances converge toward a common peak near the structural resonance frequency of 112–116 Hz, which is consistent with the resonance behavior characterized in Section 4.5.

The electrical power delivered to each load, computed from $P_{\mathrm{Load}}=V_{\mathrm{load},\mathrm{rms}}^{2}/R_{\mathrm{Load}}$, exhibits a maximum at an intermediate resistance rather than at the highest or lowest tested value. This behavior reflects the classical impedance-matching condition for a capacitive piezoelectric source. Within the quasi-static regime, the PVDF element presents an effective source impedance of magnitude $|Z_{p}|=1/\left(\omega C_{\mathrm{PVDF}}\right)$. When $R_{\mathrm{Load}}\ll \left|Z_{p}\right|$, the terminal voltage is small and the response is current dominated, yielding low power despite high current. Conversely, when $R_{\mathrm{Load}}\gg \left|Z_{p}\right|$, the voltage approaches its open-circuit value, but the current drawn becomes negligible, again resulting in reduced power delivery. Maximum power transfer therefore occurs at an intermediate resistance that balances these competing effects.

The peak power in Figure 9 occurs in the range of approximately 150–200 kΩ, coinciding with the structural resonance near 112–116 Hz. For a capacitive source, the impedance-matched load resistance is given by Equation (33) [17].

This value lies below the observed optimum of 150–200 kΩ. The four tested resistances cannot, however, distinguish an optimum at 111 kΩ from one near 160 kΩ, so the measurement is consistent with Equation (33) within the resolution of the sweep. Because the resonance frequency is independent of load, the coupling is weak and the full coupled-domain optimum converges to this quasi-static value [17]; a finer resistance sweep is identified as future work to locate the optimum precisely.

For practical implementation, a power-conditioning circuit with an input impedance in the 110–200 kΩ range would maximize energy extraction at resonance, while for off-resonance operation at 30 Hz, the corresponding capacitive-matching resistance is $1/(2\pi fC_{P})\approx 424$ kΩ.

## 5. Conclusions

This work presented a full polymer piezoelectric flextensional energy harvester (FPPFEH) in which a single-layer PVDF film was bonded to a 3D-printed PLA flextensional frame. The frame redirected an out-of-plane compressive input into amplified in-plane tensile stress in the film, enabling $d_{31}$-mode electromechanical conversion without resonance tuning. For the θ=10° prototype, the free-body model gave an ideal geometric amplification factor $M_{F}=\cot\theta \approx 5.67$; because no local force or strain measurement was performed, this value was treated throughout as an ideal upper bound rather than a validated device property.

Impedance characterization confirmed stable transducer behavior across the intended off-resonance band: the bonded film retained a nearly constant capacitance ($C_{P}\approx 12.5$ nF) and low dielectric loss over 20–50 Hz, with a resonance near 104 Hz and an anti-resonance near 114 Hz. Under off-resonance force-controlled excitation, the open-circuit voltage scaled linearly with the applied dynamic force and was nearly frequency-independent once the finite input-impedance attenuation was removed. The ideal quasi-static model reproduced this linear force–voltage dependence, together with the correct waveform and phase, but overpredicted the absolute voltage by a nearly constant factor of approximately seven. This scalar offset was consistent with the elastic compliance of the polymer frame, represented by a force-transfer efficiency $\eta_{F}$, together with the in-plane anisotropy of the uniaxially oriented film; neither quantity was measured independently, and the model was therefore reported as an upper bound rather than a validated absolute predictor.

The practical capability of the device was demonstrated rather than optimized. Under a 30 Hz, 12.32 N_rms_ excitation, the rectified output charged a 6600 μF supercapacitor to 2.10 V in 14 min, corresponding to 14.55 mJ of stored energy. In resonance-mode base excitation (0.05–1 g), the peak voltage reached 12.11 V at 1 g near the 114 Hz anti-resonance, while the response became amplitude-dependent at higher drive levels, consistent with mild stiffness softening of the polymer frame and adhesive bond line. Load-dependent measurements near resonance placed the maximum delivered power in the 150–200 kΩ range, consistent with the capacitive impedance-matching estimate within the resolution of the coarse four-point sweep.

Overall, these results establish the feasibility of a fully polymeric, additively manufactured flextensional harvester operating in both off-resonance force-driven and resonance-mode regimes. Rather than an optimized device, the FPPFEH is presented here as a proof of concept whose value lies in demonstrating that low-frequency mechanical loading can be redirected into usable axial strain and stored electrical energy through a lightweight PLA–PVDF architecture. The remaining quantitative gaps of an independently measured force-transfer efficiency and effective piezoelectric coefficient, together with finer force and resistance sweeps, define the scope of future work needed to advance the concept from this feasibility demonstration toward a quantitatively validated and optimized harvester.

## Figures and Tables

**Table 1:** Prototype geometry, material properties, and fabrication parameters.

Category	Parameter	Symbol	Value
PVDF geometry	PVDF-layer thickness	$t_{\mathrm{PVDF}}$	100μm
	Electrode-covered length	$L_{\mathrm{PVDF}}$	113.3mm
	Electrode-covered active width	$W_{\mathrm{PVDF},\mathrm{active}}$	76.0mm
	Unelectroded overhang	—	$10.0\times 113.3\;{\mathrm{mm}}^{2}$
	Bonded length at each end	$L_{\mathrm{glue}}$	12.2mm
	Mechanically active length	$L_{\mathrm{mech},\mathrm{active}}$	88.9mm
	Electrically active length	$L_{\mathrm{elec},\mathrm{active}}$	113.3mm
Electrodes	Electrode material	—	Screen-printed silver ink
	Electrode thickness, each face	$t_{\mathrm{Ag}}$	6μm
	Width-participation factor	χ	1.0
	Measured capacitance	$C_{P}$	12.5nF
PVDF properties	Transverse piezoelectric coefficient	$d_{31}$	−25pC/N
	Manufacturer permittivity	$\varepsilon_{33}^{T}$	$1.14\times 10^{-10}\;F/m$
	Permittivity inferred from $C_{P}$	$\varepsilon_{33,\mathrm{eff}}^{T}$	$1.45\times 10^{-10}\;F/m$
	Estimated Young’s modulus	$E_{\mathrm{PVDF}}$	∼2.5GPa
Frame geometry	Arm inclination angle	θ	10°
	Frame length	$L_{\mathrm{frame}}$	113.3mm
	Frame thickness	$t_{\mathrm{frame}}$	3.81mm
	Frame width	$W_{\mathrm{frame}}$	76.0mm
	Ideal geometric amplification	$M_{F}$	5.67 (=cot10°)
Frame fabrication	Material	—	PLA (FDM)
	Infill density	—	100%
	Layer height	—	0.20mm
	Estimated Young’s modulus	$E_{\mathrm{PLA}}$	2.5–3.5GPa
Bonding	Adhesive	—	J-B Weld ClearWeld Syringe Epoxy Adhesive
	Adhesive-layer thickness	$t_{a}$	<100μm
	Bonding condition	—	Flat; no prescribed pretension
	Surface preparation	—	Cleaning and light abrasion
	Cure condition	—	24 h at room temperature, clamped

**Table 2:** Summary of frequency-dependent electrical behavior of the bonded PVDF–PLA assembly.

Frequency Band	Capacitance Behavior	Loss Behavior
20–50 Hz	$C_{P}=12.5$ nF	Low tanδ
100–105 Hz	Local perturbation in $C_{P}$	Peak in tanδ

**Table 3:** Measured peak open-circuit voltage at 30 Hz compared with the ideal model prediction of Equation (20) ($\eta_{F}=1$). $V_{\mathrm{model}}=\Gamma_{\mathrm{ideal}}F_{0}$, with $\Gamma_{\mathrm{ideal}}=10.1\;V/N$, evaluated using the measured capacitance $C_{P}=12.5\;\mathrm{nF}$ and $F_{0}=\sqrt{2}F_{\mathrm{in},\mathrm{rms}}$. The $V_{\exp}$ values are as measured and uncorrected for the 7.9% input-impedance attenuation.

$F_{\mathrm{in}}$ (N_rms_)	$F_{0}$ (N_peak_)	$V_{\exp}$ (V_peak_)
3.52	4.98	5.90
6.79	9.60	12.90
9.56	13.52	18.50
12.32	17.42	24.40
19.12	27.04	29.19

**Table 4:** Resonance peak and bandwidth characteristics of the FPPFEH.

Acceleration	$V_{\mathrm{peak}}$ (V)	$f_{\mathrm{peak}}$ (Hz)	$V_{\mathrm{peak}}/g$ (V/g)	Δf (Hz)	*Q*
0.05 g	1.11	115.94	22.2	3.35	34.6
0.1 g	2.02	115.38	20.2	3.86	29.9
0.3 g	5.01	116.49	16.7	3.75	31.1
0.5 g	6.51	113.42	13.0	8.16	13.9
1.0 g	12.11	112.88	12.1	7.10	15.9

## Data Availability

The original contributions presented in this study are included in the article. Further inquiries can be directed to the corresponding author.

## Abbreviations

The following abbreviations are used in this manuscript:

FPPFEH	Full Polymer Piezoelectric Flextensional Energy Harvester
PVDF	Poly(vinylidene fluoride)
PLA	Polylactic Acid
FDM	Fused Deposition Modeling
RMS	Root Mean Square
